# Biology of colorectal cancer

**DOI:** 10.3332/ecancer.2015.520

**Published:** 2015-04-09

**Authors:** Francisco Arvelo, Felipe Sojo, Carlos Cotte

**Affiliations:** 1 Centre for Biosciences, Institute for Advanced Studies Foundation-IDEA, Caracas 1015-A, Apartado 17606, Venezuela; 2 Laboratory for Tissue Culture and Tumour Biology, Institute of Experimental Biology, Central University of Venezuela, Apartado 47114, Caracas, Venezuela

**Keywords:** colorectal cancer, metastasis, adenoma, proto-oncogenes, tumour suppressor genes, microsatellite instability, familial adenomatous polyposis, hereditary non-polyposis colorectal cancer

## Abstract

Colorectal cancer is a serious health problem, a challenge for research, and a model for studying the molecular mechanisms involved in its development. According to its incidence, this pathology manifests itself in three forms: family, hereditary, and most commonly sporadic, apparently not associated with any hereditary or familial factor. For the types having inheritance patterns and a family predisposition, the tumours develop through defined stages ranging from adenomatous lesions to the manifestation of a malignant tumour. It has been established that environmental and hereditary factors contribute to the development of colorectal cancer, as indicated by the accumulation of mutations in oncogenes, genes which suppress and repair DNA, signaling the existence of various pathways through which the appearance of tumours may occur. In the case of the suppressive and mutating tracks, these are characterised by genetic disorders related to the phenotypical changes of the morphological progression sequence in the adenoma/carcinoma. Moreover, alternate pathways through mutation in *BRAF* and *KRAS* genes are associated with the progression of polyps to cancer. This review surveys the research done at the cellular and molecular level aimed at finding specific alternative therapeutic targets for fighting colorectal cancer.

## Introduction

The colon is one of the fundamental parts of the digestive tract, as the largest and first of the segments of the large intestine, located between the small intestine and the rectum. Its principal functions are the absorption of water, minerals, and nutrients, and to serve as a storage area for the waste material that forms the feces. It consists of four sections: the ascending colon located on the right side; the transverse colon; the descending colon located on the left side, and the sigmoid colon or sigmoid. Together they constitute an irregular and thick organ because of the longitudinal disposition of muscular fibers, with a less developed submucosa, but a very evident mucosa as it is full of lymph nodes which confer its characteristic appearance. The mucosa, which is thicker than that of the small intestine, has multiple tubular invaginations called *‘crypts of Lieberkühn’*, which are wide, deep, and numerous, along the surface of its epithelium, and in which the regeneration of the epithelium takes place [1]. Because of its biological nature, the colon has a high level of cellular regeneration and a physiological role in the economy of the organism which exposes it to many agents of a physical, chemical, and biological nature, which increases the possibility of developing diverse pathologies, including cancer.

## Incidence of colorectal cancer

Colorectal cancer (CRC) is a serious health problem in the Western world, and has the third highest frequency among the tumour causing conditions which affect the inhabitants of developed and developing countries. Among males, CRC follows lung and prostate tumours, and its incidence is higher between the ages of 50 and 65. Among females it follows breast cancer, occupying the second place in terms of incidence. Around the world there are approximately one million new cases per year, affecting 550,000 men and 470,000 women, which reaffirms the importance of this pathology as a public health problem. CRC represents 13% of the causes when considering all tumours, but similar to many other cancers, there are major differences between the less and more developed countries, where they are more frequent and show a clear increasing trend, as seen in North America, Australia, Japan, and Europe [2]. Meanwhile, in the past 20 years in the United States, there has been a progressive increase in the survival and decrease in mortality because of this pathology, attributable to greater advances in surgical techniques, use of adjuvant chemotherapy, improved radiotherapy techniques, and improved campaigns for primary and secondary prevention [3]. Approximately 50% of recently diagnosed patients will progress to metastatic cancer, and there is an average survival of five years for 50–60% of patients. In 2008 the World Health Organisation (WHO) determined that nearly 600, 000 people died around the world as a result of this type of cancer [4, 5].

In general the diagnosis of this cancer occurs late, which is fundamentally because of the rapid formation of metastasis, and the high rate of transfer through the bloodstream is one of the primary obstacles in achieving a more effective treatment. As its incidence varies in different areas of the world, these geographic variations may be because of the variations in the genetic heritage of the different populations and their local dietary habits. Thus, among the risk factors are: personal and family history, the environment and dietary habits [6, 7]. There are people whose risk of cancer is two or three times higher than in the general population, and these include several groups: those with family histories of benign or malignant tumours of the intestine [8, 9]; those treated for an adenoma or a colorectal cancer [10]; women who have been treated for an epidemiologically related cancer, such as ovarian, uterine, and breast cancers prior to age 45 [11, 12]. It must be mentioned that at least 15% of CRC occur in families [13].

## Classification of colorectal cancer

CRC represents a good system and appropriate model for study of both the carcinogenesis and the molecular events involved in tumour development, which is the result of an accumulation of alterations in genes that have a role in controlling epithelial development and cellular differentiation. A study using this model allows for the collection of basic information from the formation of one or more adenomas, until their eventual transformation into metastatic cancer or not [14]. Along with this and based on the fact that both heredity and the environment contribute to the development of this type of cancer, these tumours permit the study of both somatic genetic alterations and environmental and dietary changes [15].

Three types of CRC can be distinguished by their forms of origin and expression: a) *sporadic* form, a terms which is used to differentiate tumours which appear in individuals who carry no mutation which makes them susceptible to developing this type of cancer, thus differentiating them from tumours which appear in persons who have a mutation associated with the illness and are characterised by not showing any type of family link. Nevertheless, this difference is not absolute, as the genetic factor seems to influence the likelihood of the appearance of the cancer, even in the absence of a specific mutation. The vast majority of CRC, between 60 and 80%, are of sporadic type [16]; b) the *family* type, for which no associated gene has been identified, constitutes 20–40% of the cases. Population studies show that there is a greater chance of developing this tumour when family members of primary consanguinity have suffered from sporadic colon cancer, and the risk is two to three times higher than in the normal population. Environmental factors probably determine which of the individuals who are genetically predisposed will develop this type of cancer. Nevertheless, family studies have been done which indicate that this family risk is the result of a partial *hereditary* susceptibility [17]; c) the hereditary type, with two tumour variants which can be distinguished by the predisposition to being related to the presence of adenomatous polyps or not. We can distinguish: 1) *familial adenomatous polyposis* or FAP using its initials in English, in which the patients present with multiple polyps, which in the absence of preventive surgery, one or more may become malignant at the average age of 40 [18]; 2) the variant not associated with polyposis, or HNPCC (hereditary nonpolyposis colorectal cancer) by its initials in English, which is a malignant tumour with a high risk for developing a non-digestive cancer [19]. In the case of the *sporadic* type, this includes the majority of CRC cases, representing 60–80% of them, and is characterised by the fact that it does not show any type of family relationship.

## Lynch syndrome

The hereditary syndromes of the HNPCC variant, previously known as *‘Lynch syndrome’*, give rise to the development of colorectal cancer, and constitute 3% of the total cases. It was described in 1913 by Alfred Warthin and characterised by Lynch in 1974, whose name it bears [20]. It is a pathology which is inherited in an autosomic and dominant manner, affecting men and women in the same family equally, and the genetic alteration is transmitted from parents to children without any skipping of generations.

The cancers associated with Lynch syndrome tend to affect the cecum or the right colon and constitute 70% of the 40% sporadic cases. They appear in polyps or adenomas which are large and flat, with a high degree of dysplasia, and can or cannot be fluffy [21], and possess hereditary mutations in the *MMR, MSH2, MLH1, MSH6, PMS2,* and *PMS1* repair genes. They are more frequent in cases with the genes *MSH2* and *MLH1*, constituting 90% of the total [22], and cases with mutations of the *MSH6* gene are much less frequent [23]. The tumours arise through somatic inactivation of the gene which was previously mutated in the germinal line, whether by the loss of heterozygosity or LOH by its initials in English, or by somatic mutations, or hypermethylation of promoters [24, 25]. The inactivation of these genes in the patients causes an alteration in the repetitive sequences or microsatellites which occurs at the mean diagnostic age of 45, as in the case of patients with FAP polyposis. The frequency of the formation of adenomas is similar to that of the normal population, but because of the alteration of the *MMR* genes, the rate of mutations is two to three times greater, resulting in a greater accumulation of these and a faster progression toward malignancy [26, 27].

Two types of HNPCC are defined clinically: in Type I the tumours are exclusively located in the colon; in Type II the tumours are outside the colon, located in the endometrium, ovary, stomach, hepatobiliary tract, urinary tract, pancreas, or central nervous system. Patients with HNPCC cancer develop a finite number of adenomas which may become malignant within a short time in comparison with patients with FAP, whose polyps are more diffuse. These tumours show a mucinous histology, with lymphocytic infiltration and are poorly differentiated, the characteristics which they share with the sporadic tumours having high microsatellite instability, while also being diploid type tumours that being in contrast to the sporadic tumours [28]. At the same time, HNPCC tumours show some of the characteristics of conventional adenomas, such as the presence of mutations of *APC, β-catenin (CTNNB1),* and/or *K-ras*. Lymphocytic infiltration and the coexistence of adenomas are other characteristics of these tumours [29].

In order to select the families with Lynch syndrome, the most commonly used clinical criteria are the *Amsterdam I and II criteria*, which require: a) that there be more than three members of the family with CRC cancer or tumours associated with the syndrome, such as those of the endometrium, ovary, small intestine, bile ducts, urinary tract, and others; b) that there be affected family members in several generations; c) that some of these cases have been diagnosed before the age of 50. Nevertheless, only 60% of the individuals who meet these criteria have a mutation of one of the *MMR* genes. Other clinical criteria which are used are the Bethesda criteria, which describe when a CRC is suspected of being a Lynch syndrome. It requires, among other criteria, that several cases of CRC exist in the family, regardless of age, or that there also exist a single case diagnosed before the age of 50 [30].

## Clinical varieties of HNPCC

### A) Turcot syndrome or MMR deficiency syndrome

This syndrome manifests itself in consanguineous marriages, such as those which occur between brother and sister, and is a variant of the HNPCC tumour [31] which is characterised by the coexistence of tumours of the colon (FAP or HNPCC) and the CNS (cerebral, medulloblastoma, astrocytoma, or glioblastoma) [32], for which symptoms frequently appear in the second decade of life. The prognosis for survival in these patients is very low, an average of 20 years. The existence of two subtypes of Turcot syndrome have been suggested: BTP Type I (Brain Syndrome Polyposis Type I) caused by mutations of the *MLH1* and *PMS2* genes; and BTP Type II (Brain Syndrome Polyposis Type II) when associated with mutations of the *APC* gene. According to its manifestations in the colon, Turcot syndrome is classified in three groups. a) Type 1, characterised by multiple polyps, between 20 and 10, with malignant changes: b) Type 2, characterised by at least 10 polyps of more than 3 cm in diameter with an uncertain pattern of inheritance; c) Type 3, with clinical findings similar to FAP polyposis and manifestations of colorectal carcinoma prior to the age of 30 [33].

### B) Muir-Torre syndrome

This syndrome is a variant of Lynch syndrome which is characterised by the presence of tumours in the sebaceous glands (keratocarcinomas) which are associated with one or more visceral tumours, colorectal, endometrial, and urological cancers. It appears in both sexes, with a slight predominance in males with an average age of 53, the age range being between 23 and 89 years [34]. It presents a pattern of dominant autosomic inheritance related to mutations of the *MSH2* gene [35], although it can also be because of mutations of the *MLH1* and *MSH6* genes [36, 37].

In addition, two precancerous states are recognised: *ulcerative colitis* and the presence of *adenomas*. Studies have shown that there is a risk of developing CRC in people who suffer from *haemorrhagic rectocolitis* which is further elevated when it appears at a very young age, and has an evolution of more than ten years [38]. These situations are the most frequent, and the most severe form is found in patients who have had a total colectomy, as well as with *Crohn’s disease*, which is also associated with a high risk of colon cancer [39]. In addition, adenomas—commonly called ‘*polyps*’—are a common pathology which are characterised as pedunculated benign epithelial tumours [40, 41]. In countries with a high incidence of colon cancer there is a prevalence of adenomas which varies between 30 and 60%, a fact demonstrated through autopsy performed on subjects over 55. The adenoma/cancer relationship rests on epidemiological, clinical, anatomopathological, and genetic studies. All this to be considered along with the appearance of the adenomas, geographical distribution, the transformation of an adenoma into cancer in patients who have not had surgery, in extended polyposis, sequelae because of adenomatosis, and through the study of the accumulation of changes as related to the increase in the grade of dysplasia of adenomas until they become cancerous [42].

## Genetics of colon cancer

*Adenoma-carcinoma sequence.* In the carcinogenesis of CRC, several means by which the normal colon cells can become malignant have been described, and studies have centred on the genetic changes which are found in three fundamental categories of genes: 1) *tumour suppressor genes* or *TSG*, such as *APC, DCC, TP53, SMAD2, SMAD4 and p16INK4a)*; 2) protooncogenes, such as *K-ras, N-ras*; 3) DNA repair genes, such as *MMR* and *MUTYH*) [43]. The first means described is the *suppressor or chromosomal instability*, recognised in the Fearon and Vogelstein genetic model [44], which includes FAP tumours and in 80% of sporadic tumours. Each tumour created by changes in this means develops *chromosomal instability* or CIN, with frequent cytogenetic changes and loss of allelic heterozygosity [45, 46]. This model proposes that the histopathological sequence of the progression of CRC is because of mutations in concrete genes, principally tumour suppressor genes. The sequence of changes is initiated by the mutation or loss of the *APC* gene (5q21-q22), followed by mutations on *KRAS* (12p12.1), and the mutations on *TP53* (17p13.1) and *DCC* (18q21.3). The *APC* gene is mutated in 70–80% of colorectal tumours, with 50% showing independent mutations on the β-catenin, indicating the preponderant role of the Wnt as a means in the control of colorectal tumourigenesis, above all in its initial stages [47, 48]. The mutations on the *K-ras* oncogene are produced during an even more advanced stage, preferentially affecting codons 12 and 13, and are found on up to 40% of colorectal tumours [49]. The *DCC* gene is located on chromosome 18q21 and has a function in CRC which is still not well understood, but is made up of 28 or 29 exons which code for a protein of 1.147 amino acids which cross both sides of the cell membrane. Its extracellular region is homologous to that of the cellular adhesion proteins, which have extracellular domains similar to the immunoglobulins [50]. The *DCC* protein may affect the epithelial/mesenchymal interactions, perhaps regulating processes of proliferation and/or differentiation. Its expression is reduced or almost nonexistent in more than 50% of CRC, and different mutations have also been detected [51]. The fact that the *DCC* gene may be considered a tumour suppressor was confirmed by a study in which the tumour phenotype was reverted by transfection of the *DCC* gene [52].

The p53 protein intervenes in the control of the cellular cycle, replication and repair of the DNA maintaining genomic stability, activating apoptosis, and participating in the cellular response to noxious agents. This protein is tetrameric and made up of four sub-units, each of these with 393 amino acids [53], its proteic overexpression precedes the malignant change in the majority of human cancers, including CRC [54]. The *p53* gene is located on chromosome 17 and has 20kb, as it contains 11 exons and codes for a nuclear phosphoprotein of 53 kDa, as it is a transcriptional regulator which acts as a brake on the G1/S control point and intervenes in multiple functions such as: the activation of the stopping of the cellular cycle, senescence, differentiation, and apoptosis [55].

The Fearon-Vogelstein model is still considered valid for illustrating the concept of *‘multiple steps’* of tumour progression, although one has to understand that the sequence of proposed changes is the result of a statistical analysis. For this reason, changes in the tumours of different patients group to form a single model of the process was proposed, although this does not imply that an individual has to show all of the changes. This model was completed in 2002 with the discovery of the MAP kinase [56, 57], which affirms that the pattern of mutations of the *MUTYH* gene is derived from a deficiency of the proteic function, which is characterised by an excess of G→T transversions in GAA sequences, which are susceptible to provoking the appearance of stop codons [58]. Because of the fact that *APC* contains an elevated number of these sequences, the mutations on *MUTYH* increase the rate of somatic mutation of *APC*, which in turn initiates neoplastic transformation [59].

The second means is by *mutating* or *microsatellite instability*, MSI by its initials in English, which includes the *Lynch syndrome* tumours and approximately 15% of the sporadic tumours [60]. This explains the appearance of CRC in the cases of deficiency in the system for mismatch error repairs, MMR (*mismatch* repair or *base unpairing*), in which the mutations on the *MMR* genes provoke a state of genomic instability which leads to the appearance of a *hypermutator* phenotype, also known as *microsatellite instability*, MSI [61]. This phenotype is produced because the alteration of the *MMR* genes favours the appearance of new mutations, predominantly in repetitive sequences, *microsatellite loci* of AA[S]NAA or CA[CA]NCA type, generating an instability in the microsatellite regions which expand or contract with the insertion or deletion of repetition units. This causes the inactivation of various types of genes, such as those which regulate apoptosis, as in the case of the *BAX* or *Caspasa-5* genes [62, 63] and the genes implicated in the control and regulation of cellular growth, such as *TGFβRII, WISP-3* or *IGFIIR* [64, 65] or even *MMR* genes such as *MSH3* or *MSH6* [66].

Furthermore, the epigenetic processes have been described as one of the alternative mechanisms of carcinogenesis, even though they do not imply any genetic changes. Today ‘*epigenetic silencing*’, which in English is CIMP (initials of CpG island methylator phenotype), or *phenotype methylator means*, is recognised as the third means of the Knudson Model of colorectal tumourigenesis [67, 68]. These epigenetic changes create instability in the genes as a result of the inactivation of *TGS*, or *MSI* or *CIN* repair genes [69]. There are two types of epigenetic changes: one may modify the methylation, so that some DNA nucleotides are changed by the addition of a methyl group, -CH3, to the base. Methylation is associated with the inactivation of a particular region of the DNA; the other change may modify the acetylation, in which the histones around which the DNA spirals are changed by the addition of acetyl groups, -CH3CHO. This change weakens the interaction between the DNA and the histones, which is associated with greater genetic expression [70]. Agents with the capacity to inhibit Wnt signaling have been evaluated, as is the case of acetyl transferase, which inhibits the activity of the porcupine which is necessary for the synthesis of Wnt [71]. Other agents have also been looked into, for e.g. the non-steroidal anti-inflammatories aspirin, sulindac, celecoxib, nimesulide, piroxicam are cyclooxygenase inhibitors which show chemoprotective effects against cancer [72, 73]. The NSC668036 and FJ9 molecules, compounds which have been proven in preclinical studies, act as a key target in Wnt signaling, as is the dishevelled protein [74, 75]. Vitamin D has also been used in treatment for the prevention of CRC, as it assists the detoxification of the bile acids which activate the cancer, and which are released during the digestion of foods with a high fat content [76].

The causes which activate the hypermethylisation process are not clear, although the variability of the methylation rate in the different types of tumours shows that this mechanism is not activated randomly, rather it follows a pattern which has not yet been established. It is known that environmental factors are such as lesions due to chemotherapeutic agents, the ingestion of folates, but the actual reason or the genetics which regulate this phenomenon are not well known [77]. Nevertheless, the existence of four different genetic means for carcinogenesis of the colon have been described: 1) the Wnt/β-catenin means, associated with the adenoma-cancer sequence; 2) the means of microsatellite instability because of mutation or hypermethylation of the *MMR* genes; 3) the ulcerative colitis/dysplasia/cancer means not associated with an *APC* mutation or the formation of polyps; 4) the means of frequent hypermethylation in sporadic cancers [78].

## Wnt Wnt/β-catenin pathway-carcinogenesis

The importance of this pathway can be seen from the studies which show that 90% of CRCs develop mutations in some of their components [79]. Mutations of the *APC* and *CTNNB1* genes, which are very important in this pathway, create a resistance to the degradation of β-catenin by means of the *ubiquitin-proteasome system,* through which it accumulates in the cytoplasm and is transferred to the nucleus, where it acts to promote the overexpression of oncoproteins [80]. This creates a hyperproliferative phenotype which favours the mutations of other genes, allowing for early progression to adenoma. It is believed that the activation constituted by the Wnt/β-catenin pathway is not only important in the initiation of colorectal carcinogenesis, but that it may also control the malignant potential of the cells at more advanced stages, and may be a target for the development of new CRC therapies [81].

This pathway also participates in the processes of regulation, differentiation, proliferation, and cell death, as it is involved with numerous abnormalities of embryonic development, growth, and homeostasis. The Wnt proteins act as ligands to stimulate the signal transduction pathways mediated by receptors in vertebrate and invertebrate organisms [82]. Presently four Wnt signaling pathways are known: 1) the canonic or Wnt-β-catenin pathway; 2) the Wnt/Ca+2 pathway which involves the kinase A protein; 3) the planar cellular polarity pathway; 4) the pathway which includes the kinase C protein and intervenes in the process of myogenesis.

Some of the Wnt proteins activate and modulate the canonic pathway, however, the most important and most studied is the cytoplasmic control and regulation pathway related to the β-catenin protein [83]. The increase in cytoplasmic β-catenin permits its entry into the nucleus, where it activates the transcription of genes whose protein products participate in processes of cellular division, embryonic development, and morphogenesis. The Wnt-β-catenin pathway is interrelated with a significant number of cellular signaling pathways, such as Notch, Hedgehog, Rac/*K-RAS,* and mTOR, which coordinate the development of organs and maintain the homeostasis of some tissues [84]. The growth factor of FGF fibroblasts and the transformative growth factor beta TGF-β also interact with Wnt-β-catenin to regulate their activity and control specific cellular processes. These pathways rarely function alone or in an isolated manner, and the erroneous activation of one pathway in particular may result in the development of a cancer [85].

It must be emphasised that the pathways play preponderant roles in the maintenance of the homeostasis of different tissues such as the intestine, breast, skin, blood, and brain, in addition to regulating the somatic stem cell niches. The abnormal regulation of these pathways give rise to the neoplastic proliferation of the tissues indicated, apart from other pathologies which originate as a result of changes in the Wnt pathway; but most studies focus on their relationship with cancer. Of the four known Wnt signaling pathways, the Wnt-β-catenin pathway has been identified as the principal one responsible for cellular changes which cause cancer. Various regulator genes for this pathway are known, and are found with changes in different types of human cancers, while the common denominator is the modification of the expression of the Wnt-β-catenin genes [86]. The implication of β-catenin in malignant processes was first known for CRC, as this protein forms a complex with the APC protein, whose participation in carcinogenesis was discovered in studies of hereditary cancer caused by familial adenomatous polyposis. It is noted that 80% of the cases of sporadic colon cancer, as well as the hereditary forms, are caused by mutations of the *APC* gene [87]. Patients with familial polyposis inherit the defective *APC* allele at an early age, which causes the formation of numerous adenomatous polyps in the colon, which results in the clonal growth of epithelial cells in which the second *APC* allele is made inactive. The adenomas give rise to the appearance of adenocarcinomas as a result of the accumulation of mutations of oncogenes or tumour suppressor genes such as *KRAS, p53,* and *Smad4*. The absence of *APC* causes destabilisation of the β-catenins, which leads to the transformation of the epithelial cells [88].

## E-cadherin and α-catenin

Infiltration and metastasis require multiple properties and mechanisms, among which the reduction of the capacity for intercellular adhesion in the primary tumour at the initial stages stands out [89]. In this invasive capacity they play a fundamental role in the molecules of cellular and tissue adhesion, which are distinguished into four structural classes: the immunoglobulin family; the selectins, the integrins, and the E-cadherin [90, 91]. The latter is part of a protein complex, the E-cadherin–catenin, which associates the α, β, γ catenins with the actin filaments and other proteins such as APC [92]. The human *α catenin* gene is located on chromosome 5 in the 5q21-22 region, while the *β catenin* is located on chromosome 3 in region 3p21.

The β and α catenins bind with the recently synthesised cytoplasmic cadherin before being transported toward the cell membrane [93]. The gene for the E-cadherin protein is located on chromosome 16, in region q22.1, in an area which is often found with alterations in cancer cases [94]. Studies of colonic type adenomas with severe dysplasia and colon cancer show a progressive reduction of the expression of E-cadherin [95]. This suggests a change in its expression during the early stages of carcinogenesis of the colon, as when comparing them to more undifferentiated tumours, they show a greater expression of E-cadherin and α-catenin [96]. The level of expression of ARNm of the E-cadherin is correlated with the prognosis in CRC [97]. In other studies, the capacity of β-catenin to form complexes with the APC protein has become evident, thus establishing a likely competitive relationship between APC and E-cadherin to join with β-catenin [98]. The majority of colon cancers show a modification of the APC or β-catenin protein, which suggests an important role in the initiation of carcinogenesis of the colon [99].

## The adenomatosis polyposis coli gene

*Familial adenomatous polyposis*, FAP, is responsible for one percent of CRCs, and is a hereditary illness with dominant autosomic characteristics. Its principal clinical manifestation is the existence of multiple colonic and rectal adenomas which may exceed 100, which clearly indicates a diagnosis of FAP polyposis. Among these adenomas, usually one or more will become malignant in a nearly constant manner before the age of 40, while presenting associated clinical manifestations, such as duodenal adenomas, fundic gland polyps, or bone, subcutaneous, and desmoid tumours [100, 101]. The genetic study of families with FAP has permitted the identification of the *APC* gene as responsible, which located on chromosome 5q21, suffers deletions and various types of early mutations which are called *‘nonsense mutations*’, *‘frameshift mutations’* and *‘missense mutations’* [102]. FAP polyposis is related to a mutation of one of the two alleles, both in the germline and in all of the tissues of the organism. In the CRC cells, one of the two alleles may be lost or may show a somatic mutation [103]. This gene shows an identical mutation in more than 60% of cases of sporadic cancer, indicating that the mutations that are evident in FAP are comparable to the mutations of the sporadic forms. Within this problem we must consider that another possible mechanism may be the hypermethylisation of the region which promotes the *APC* gene, which may lead to a reduction in the transcription of the gene [104, 105].

We must always consider the fact that the predisposition to suffer neoplastic illnesses depends on endogenous and exogenous factors which finally modify the expression of specific genes [106]. The *genomic imprint* is the pattern of different methylations of homologous genes according to the maternal and paternal origins of the chromosome. This concept may explain the differential expression or lack of expression of a certain allele, given that we know that the methylated genes are inactive, while the non-methylated or hypomethylated genes may be transcribed to generate a protein product [107, 108]. The epigenetic phenomena associated with CRC do not include changes in the DNA sequence, but potentially reversible genetic modifications which lead to genomic instability [109]. Three types of altered DNA methylation patterns are found in human cancers, which are: hypomethylation, hypermethylation, and loss of imprinting or LOI [110]. The LOI pattern refers to the loss of the differential expression of parental alleles, which is dominant in embryonic tumours [111]. Hypomethylation of the DNA, the hypermethylation of the tumour suppressor genes, and the inactivation of *miARN* genes because of DNA methylation have also been described in human tumours [112].

The *APC* gene codes a notably hydrophobic protein with a molecular mass of 311.8 kDa [113], which presents a diffuse cytoplasmic distribution accumulating principally along the lateral or sub-apical margins of the cells [114]. The most common form loses the 10ª exon and codes for an APC protein with 2843 residues which are divided into various *domains*: a) of oligomerisation which is localised in the N-terminal region of the protein and consists of seven repetitions of 6–57 amino acids [115]; b) an *armadillo* region in the amino-terminal end which is highly conserved, located between amino acids 453 and 767 which allows it to play a role in the stabilisation and motility of the cytoskeleton [116]; c) a domain formed by a group of repetitions of 15 to 20 amino acids in their central portion and carboxy-terminal end which contains a basic domain and joining sites for other proteins. The APC protein presents a domain of seven motifs of 20 repeated and highly conserved amino acids, located between residues 1262 and 2030. This domain confers the APC protein with joining sites for β-catenin [117]; d) a domain of repetitions of 15 amino acids which is highly conserved and located between amino acid 1020 and 1169 which encompasses three repetitions of 15 amino acids which function as joining sites for β-catenin [118]; e) a basic APC domain located in region C-terminal amino acids 2200 to 2400 and seems to function as a domain for joining with the microtubules [119].

The APC protein is known to have six regulatory functions and one blocking function [120], which are: 1) regulation of the levels of β-catenin and thus the signals induced by the same [121]; 2) regulation of cellular adhesion through β-catenin and E-cadherin [122]; 3) regulation of cellular migration and chromosomal stability through interaction with the microtubules [123]; 4) regulation of neural function. The hDLG (human homologue of Drosophila discs large tumour suppressor) protein is habitually located in the epithelial cells, found between the cell–cell unions [124]; 5) regulation of cellular motility. The APC regulates cellular polarity and migration through the control of the actin cytoskeleton [126]; 6) that of blocking the cellular cycle, which possibly occurs through direct inhibition of the components of the cellular cycle and apoptosis [125].

## BRAF, NRAS, VEGF genes

*BRAF gene—*is located on the long arm of chromosome 7 (7q34) and codes a cytoplasmic serine/threonine kinase of the RAS family which mediates the transduction of signals on the MEK/ERK route, which is an important conduit for cellular growth, differentiation, and regulation, as well as the promotion of apoptosis. In some CRCs the *BRAF* oncogene is found to be mutated, and the mutations are associated with a CpG methylation region [127]. Several early, somatic, or activating mutations in the *BRAF* oncogene cause the protein to become hyperactive, releasing a cascade of signals which may play an important role in some specific malignant tumours. Approximately 90% of the *BRAF* V600E mutations are known, involving the substitution of glutamic acid (E) by valine (V) in position V600 of the protein chain, resulting in a constitutionally active *BRAF* oncogene [128]. As a result of this activation, the hyperactive MEK and ERK signaling lead to excessive cellular proliferation and survival, which is independent of the growth factors. The *BRAF* oncogene signaling may lead to an uncontrolled increase in cellular proliferation and resistance to apoptosis [129]. Approximately 30–50% of CRC tumours have a mutated *KRAS* gene, which indicates that up to 50% of the patients with this type of cancer could respond to therapy with antireceptor antibodies against the epidermal growth factor, EGFR. Nevertheless, from 40–60% of patients with wild-type *KRAS* tumours do not respond to such therapy [130]. In these patients the data suggest that the mutated *BRAF* gene, present in 5–10% of the tumours, may affect the response to these agents. It is not clear to what extent the lack of response in the wild-type *KRAS* gene is because of *BRAF* mutations, but the data suggest that this mutated oncogene confers resistance to anti-EGFR therapy, determined to go beyond first line treatment [131]. In a study of 524 patients with *BRAF* mutant and wild-type *BRAF* colorectal cancer, the survival for the first group was 10.4 months, while that for the second group was 34.7 months. The *BRAF* mutation activates the MEK/ERK pathway by means of its effectors, which are the production and promotion of the malignant phenotype through genetic expression and proliferation [132]. Nevertheless, the clinical results with *BRAF* inhibitors in CRC tumours have been quite deceptive. At the same time, in a study of CRC cells with the mutated *BRAF* phenotype and resistant to vemurafenib, it was revealed that the inhibition of *BRAF* by this compound produced a rapid activation of the EGFR growth factor receptor, which stimulated continuous proliferation in the presence of the *BRAF* inhibition [133]. It was also demonstrated that the suppression of EGFR by cetuximab, erlotinib, gefitinib, and vemurafanib causes an inhibiting effect on CRC with the *BRAF* mutation in both *‘in vitro’* and *‘in vivo’* models, results of which indicate that combination therapy with *BRAF* and EGFR inhibitors may be more effective. This study, in which vemurafenib and erlotinib were combined, resulted in the regression of tumours formed by xenografts using cell lines of colorectal cancer and the reduction of the Ki67 proliferation marker [134].

*RAS gene—*The *RAS* family of genes: *HRAS, NRAS,* and *KRAS* constitute one of the most frequently altered groups of oncogenes in human neoplasias [135]. For example, the *KRAS* oncogene participates in the signaling of the PI3K/PTEN/AKT and RAF/MEK/ERK pathways [136, 137], consequently *KRAS* mutations constitute about 85% of all of the *RAS* mutations in human tumours, while *NRAS* mutations make up approximately 15%, and *HRAS* account for only 0.12 to 1% [138]. The *RAS* gene seems to be specific for tumours of the colon, pancreas, and lung, which have a high frequency of *KRAS* mutations. The proteins encoded by these genes have a 21Kd (p21) protein structure which has GTPasa activity, acting on the signal transduction pathways for cellular growth and differentiation [139]. The mutation of this gene is the most common genetic event observed in the development of malignant human tumours, at a rate of 30% in the lung, 40% in the colon, 80% in the pancreas, and 55% in the thyroid [140]. About 90% of the mutations of this gene are located on specific sites of the first exon, approximately 80% on codon 12; and on codon 13 their frequency is 15–20% [141]. On the second exon they are located on codon 61, which has a frequency of mutation of less than 3%, but the most frequent mutation occurs on the second nucleotide of codon 12 and predominantly corresponds to guanine–adenine transitions with the substitution of aspartic acid by glycine.

The therapy used in some advanced stages of colon and rectal cancer include the use of monoclonal antibodies such as panitumumab and cetuximab, which can block the activation of EGFR [142]. In patients who do not respond to this therapy, it has been demonstrated that the tumour cells carried one of the mutations of the *KRAS* gene, which is found located on the *EGFR* gene pathway, which caused the activation of this pathway independently of the EGFR blocking [143]. At the same time, it was demonstrated that the *KRAS* gene, both in its wild and mutated state, was capable of predicting both the response and its utility in the tumour against the use of EGFR inhibitors [144]. Various studies have shown that some specific types of *KRAS* mutations are related to survival, as is the G12V mutation, which is associated with a more adverse prognosis for the disease in comparison with other types of mutations [145, 146].

*VEGF gene—*The chromosome location of the human *VEGF* gene is 6p21.3, and it consists of eight exons and seven introns, with a coding region approximately 14kb in length [147]. Five isoforms are generated by this gene through alternative splicing which has a size of 121, 145, 165, 189, and 206 amino acids [148]. The majority of the cell types which express this gene in both normal and pathophysiological situations express the *VEGF*121 and *VEGF*165 isoforms, while *VEGF*189 is also found in some cell types [149]. The survival and neoplastic expansion of the clone of cells are compatible with the surrounding tissue which sustains and favours these conditions, thus the stroma associated with the tumour actively sustains the progression of the CRC. Among the cells associated with the tumours are the endothelial cells and pericytes, which form the neo-vascularisation [150]. The cytokines and growth factors produced by the tumour cells create optimal conditions for growth within the microenvironment of the tumour, while the cytokines secreted by stroma cells may influence the malignant behaviour of the cells. The pro-inflammatory IL-4 and IL-1 cytokines, the growth factors such as VEGF and TGF-β-1, -2, -3, and their receptors [151] promote the transcription of genes to activate various signaling pathways which collaborate in survival, tumour expansion, and metastasis [152].

Hypoxia is the principal regulator of the expression of the *VEGF* gene both *‘in vitro’* and *‘in vivo’*, as the reduction of the pressure from oxygen causes an increase in its transcription by means of the HIF1 (*hypoxia inducible factor 1*) transcription factor [153]. Hypoxia represents a decisive stage in tumour progression, as the HIF1 transcription factor cooperates with the IL-6, TGF-β and VEGF factors in the promotion of tumour growth [154]. In colon cancer, the interaction of the levels of interleukin-6 (IL-6) and the increase of hypoxia-inducible factor (HIF)1α favours the expression of the pro-angiogenic VEGF isoform, which contributes to tumour proliferation, the escape from apoptosis and the migration of the tumour cells, to which is added its affinity with the stage of the tumour [155]. A clinical trial with a vascular endothelial growth (VEG) inhibitor, bevacizumab, demonstrated that the incorporation of this agent into standard chemotherapy for the treatment of metastatic CRC increased survival in patients with CRC cancer [156]. The 5-fluorouracil (5-FU) along with bevacizumab have been two of the principal chemotherapy agents in the treatment of CRC [157]. In the last 15 years the pattern of treatment of metastatic colorectal cancer has been based on the use of cytotoxic drugs such as irinotecan and oxaliplatin and the monoclonal antibodies bevacizumab and cetuximab [158, 159].

## Metalloproteases and colorectal cancer

The extracellular matrix constitutes a physical barrier, for which reason the correct functioning of the pathways which regulate its morphogenesis, development, tissue damage, and remodeling is essential to maintaining its integrity. Nevertheless, when some type of change is detected in said regulation, these are related with some illnesses, among which cancer stands out. The two biological mechanisms responsible for the malignancy of cancer are infiltration and metastasis [160], in which the invasive processes which occur because of the rupture of intercellular junctions caused by the proteolytic activity of the proteases play an essential part. These have the property of breaking the molecules of the extracellular and adhesion matrices, where the MMPs metalloproteases or human matrixins are found, and which can be classified as: the MMP-1 and MMP-13 collagenases; the MMP-2 and MMP-9 gelatinases; the MMP-3, MMP-10, and MMP-11 stromelysins; the MMP-7 and MMP-26 matrilysins, as well as the transmembrane types MMP-14, -15, -16, and -24 [161].

These enzymes and their levels of transcription are strictly regulated positively and negatively by molecules such as cytokines, growth factors, and tumour necrosis factors. Even the cell–matrix or cell–cell interactions are capable of modulating the transcription of the MMP proteases. In addition, there is another level of regulation in terms of their enzymatic activation by post-transcriptional modification which requires the elimination of the propeptide at the N-terminal end of the protein. Furthermore, there are tissue-specific MMP inhibitors [162]. The transcriptional regulation of these proteins is also performed by cytokines and growth factors which also control the expression of the proteases [163, 164].

The family of MMP enzymes is capable of degrading all of the components of the extracellular matrix: collagens, laminins, fibronectins, vitronectins, proteoglycans, etc. The proteases are implied in a multitude of physiological and pathological processes in which their proteolytic activity is involved. In addition, they are included in many other processes, both physiological, such as differentiation and apoptosis, and pathological, above all tumour metastasis [164]. In CRC various members of the MMP family have been well studied, in which MMP-1 is associated with a worse prognosis as it favours haematogenous metastasis [166]. A greater expression of MMP-13 has also been associated with CRC tumours with a worse prognosis [167]. The expression of MT1-MMP and MMP-14 is elevated in tumours of the colon and has been described as a target of the Wnt pathway [168] MMP-2 is related to tumour invasion and it has been demonstrated that there is a greater expression of its transcription on the invasive front of the colon tumour. In T cells, the induction of MMP-2 and MMP-9 are achieved through their incubation with WNT1 and WNT3a. Both are targets of β-catenin and present connection sites for LEF/TCF [169]. It was found that the expression of MMP-3 in MSI-L/MSS CRC tumours was greater than in MSI-H tumours, in which the levels of active MMP-9 were also lower, possibly because of a lower synthesis of MMP-3 [170]. As for MMP-7, it is related to invasion and metastasis in CRC, as well as a positive correlation in terms of its expression with β-catenin [171]. In fact, it is known that MMP-7 is a target of the Wnt pathway, given that it possesses connection sequences with TCF-4 in its promoter which respond to the presence of β-catenin. There are other molecules involved in this regulation, such as *K-ras*, which may be playing a synergistic role with the Wnt pathway [172].

## Stem cells in colorectal cancer

Stem cells are defined by two fundamental biological properties, these are *self-renewal* and *multipotency*. The first is the capacity of the cell to perpetuate itself for a long period of time, while the second is the capacity to generate all of the differentiated cells of the tissues of origin [173]. In the superficial epithelium of the mucosa of the colon there is a constant renewal, which is normal and occurs through the proliferation and differentiation of the stem cells which are found at the base of each of the *crypts of Lieberkühn*. The matured differentiated cells normally lose their capacity to divide and finally die from apoptosis [174]. Within this reality the *‘theory of cancer stem cells’* was born, which suggests that the tumours are created and maintained by a small sub-group of undifferentiated cells which are capable of self-renewal and differentiation. The theory of cancer stem cells was originally proposed by Cohnheim in 1875, and was based on four principles: 1) external or internal aggressions caused by physical, chemical, or biological factors can cause genetic damage in the stem cells; 2) the malignant stem cells create the tumours; 3) within a tumour all of the cells have the same profile; 4) the different tumours originating from different stem cells have different genetic and biochemical profiles [175]. This theory was originally based on studies of leukaemia [175], but now it has validity for CRC and is being extensively investigated for other neoplasias. Recent studies have found that stem cells for colon cancer can be identified using certain cellular markers such as CD44, CD133, CD166, and EpCAM [177, 178, 179]. Self-renewal is regulated by various pathways for both normal stem cells and malignant stem cells. For leukaemias and colon cancer, a possible common pathway is through Wnt [180, 181]. Other possible alternatives with implications for CRC include Notch, PTEN / AKT, p53, and Bmi [182, 183, 184]. The stem cells implicated in the development of cancer have important implications for prevention and treatment, as the great majority of cytotoxic agents used for treating colon cancer are designed to eliminate the actively proliferating cells. This means that stem cells implied in cancer are being obviated, which contributes to the selection of resistant cells, so that in the future the search for specific biomarkers for stem cells implied in cancer will contribute to improving the diagnosis, prognosis, and therapy for the prevention and treatment of cancer.

## Conclusion

The development of CRC can begin through the inactivation of tumour suppressor genes, which can be caused by either mutations or hypermethylations of said genes. There are also oncogenes which can suffer mutations and the genes which participate in the control of cell proliferation and apoptosis. An accumulation of multiple mutations has been found in colorectal tumours, but these are not always the same, for which it seems that the total accumulation of these mutations is more important than the order of appearance of the phenotype. In addition, the process of colorectal carcinogenesis is not unique, and there are probably several of these for the beginning, development, and progression of this type of tumour. This review shows that it is very important to further develop research on this type of cancer, as through deeper study we can uncover and understand its mechanisms, processes, and interrelationships in depth in order to find better and more specific alternatives for therapeutic targets which will allow us to optimise the treatments for this type of cancer, which is so common and so severe.
